# Professional Magnanimity

**Published:** 1848-06

**Authors:** 


### PROFESSIONAL MAGNANIMITY.

Among other changes made by the Provisional Government of France, we discover by the public prints that Orfila has been removed from the station of Dean of the Faculty, which he has held so many years with distinguished honour to himself and the profession, as well as to the country of his adoption. It is with much pleasure, however, that we have read the following account of the deportment of his successor, Bouillaud, on the occasion, communicated by the correspondent of the London Medical Times, and published in that journal of the 11th of March.

*Appointments.*—The Dean of the Faculty of Medicine, Professor Orfila, has been removed from his office by the Provisional Government, and Professor Bouillaud is appointed in his place. M. Orfila has rendered signal services to the School of Medicine, and his popularity is not diminished by the decision of Government: he received the warmest thanks of the faculty on his retirement. His succcessor, Professor Bouillaud—who was the first to acknowledge the obligations of the French medical profession to the late dean, and who moved the vote of thanks—was received by the pupils at first with some slight manifestations of displeasure, which were soon changed into unanimous applause when he explained, in a spirited address, that the honour now conferred upon him was not of his seeking, and that he had accepted of it only on condition that the choice of Government would be sanctioned by his colleagues and by the pupils of the school.

